# Cremation

**Published:** 1886-02

**Authors:** 


### Cremation.

The completion of the crematorium in Buffalo has incited a lively discussion of the proper methods of disposing of the dead. The discussion has been confined principally to the pulpit and the popular press, the medical profession manifesting an indifference which is almost surprising.

Physicians were the prime movers in the enterprise, and are the principal stockholders to day, yet we seriously doubt if one-tenth of these stockholders will ever be incinerated. From a sanitary point of view, there can be no objection to cremation. We are aware that it has been urged that cremation might tend to conceal crime, but this would happen infrequently, and it is an objection which could be overcome.

The clergy, however, claim that cremation violates our religious sentiments, and the clergy still have mighty influence in the world, although science has, seemingly, many times forced them to the wall. A custom which has been handed down thousands of years, and thoroughly interwoven with our social and religious systems, will not be set aside without good reasons. The ancient Greeks and Romans, after practicing inhumation for centuries, adopted cremation, but for very different reasons from those urged at the present day. According to the ancient Roman law, lands on which bodies were buried became *res sacræ extra commercium*—that is, sacred, and not subject to sale or taxation. The whole country was thus in danger of becoming consecrated and unproductive. The Ancients, too, regarded fire as a deity. Vesta was the purest of the goddesses, and to be consumed by her element, was looked upon as a purifying process. For these reasons, cremation became generally adopted, though not universal; for, criminals and unteethed infants were still buried, thus rendering the change to general inhumation in the time of the Christian Empire easy.

The Christian associates fire after death with a very different state of affairs. Among the Ancients, religion aided the cremationists; among the Moderns, religion opposes them. Here, as on many previous occasions, science, in the Liberal, proposing innovations; religion, in the Conservative, claiming that present customs are good enough. Science says, "the earth moves;" religion desires incontestable proofs before she believes it, and is not particularly anxious to investigate the subject either.

We must admit, however, that there is not, as yet, any crying need for cremation in America. Land is cheap and abundant near our cities. There is no necessity for overcrowding cemeteries. The soil and the vegetation over it can dispose of the products of decomposition in a harmless manner. There are few, if any, well authenticated cases where cemeteries have injured the public health. But in Europe a very different state of affairs prevails. Land for burial purposes can scarcely be obtained. Dwellings are built next to crowded churchyards. The poor have no graves of their own, but are laid in trenches, or are thrown into pits, in a manner disgusting, when compared with cremation. It is in those countries that cremation is making the most rapid progress, because a reform is there most needed.

In the United States, numerous crematories are being built, and will, in time, be put to a good use. The innovation meets much opposition, so that none of the present generation will live to see cremation generally adopted. The cremationists are doing a good work for posterity, but we greatly fear that they will see no returns from the money they have invested for a century or two.

